# Humanin Protects RPE Cells from Endoplasmic Reticulum Stress-Induced Apoptosis by Upregulation of Mitochondrial Glutathione

**DOI:** 10.1371/journal.pone.0165150

**Published:** 2016-10-26

**Authors:** Douglas Matsunaga, Parameswaran G. Sreekumar, Keijiro Ishikawa, Hiroto Terasaki, Ernesto Barron, Pinchas Cohen, Ram Kannan, David R. Hinton

**Affiliations:** 1 Department of Pathology and Ophthalmology, USC Roski Eye Institute, Keck School of Medicine of the University of Southern California, Los Angeles, CA, United States of America; 2 Arnold and Mabel Beckman Macular Research Center, Doheny Eye Institute, Los Angeles, CA, United States of America; 3 Leonard Davis School of Gerontology, University of Southern California, Los Angeles, CA, United States of America; Duke University School of Medicine, UNITED STATES

## Abstract

Humanin (HN) is a small mitochondrial-encoded peptide with neuroprotective properties. We have recently shown protection of retinal pigmented epithelium (RPE) cells by HN in oxidative stress; however, the effect of HN on endoplasmic reticulum (ER) stress has not been evaluated in any cell type. Our aim here was to study the effect of HN on ER stress-induced apoptosis in RPE cells with a specific focus on ER-mitochondrial cross-talk. Dose dependent effects of ER stressors (tunicamycin (TM), brefeldin A, and thapsigargin) were studied after 12 hr of treatment in confluent primary human RPE cells with or without 12 hr of HN pretreatment (1–20 μg/mL). All three ER stressors induced RPE cell apoptosis in a dose dependent manner. HN pretreatment significantly decreased the number of apoptotic cells with all three ER stressors in a dose dependent manner. HN pretreatment similarly protected U-251 glioma cells from TM-induced apoptosis in a dose dependent manner. HN pretreatment significantly attenuated activation of caspase 3 and ER stress-specific caspase 4 induced by TM. TM treatment increased mitochondrial superoxide production, and HN co-treatment resulted in a decrease in mitochondrial superoxide compared to TM treatment alone. We further showed that depleted mitochondrial glutathione (GSH) levels induced by TM were restored with HN co-treatment. No significant changes were found for the expression of several antioxidant enzymes between TM and TM plus HN groups except for the expression of glutamylcysteine ligase catalytic subunit (GCLC), the rate limiting enzyme required for GSH biosynthesis, which is upregulated with TM and TM+HN treatment. These results demonstrate that ER stress promotes mitochondrial alterations in RPE that lead to apoptosis. We further show that HN has a protective effect against ER stress-induced apoptosis by restoring mitochondrial GSH. Thus, HN should be further evaluated for its therapeutic potential in disorders linked to ER stress.

## Introduction

Age-related macular degeneration (AMD) is the leading cause of blindness in individuals older than 65 in developed countries. In 2004 it was estimated to affect 1.75 million adults in the United States, and is expected to affect 50% more people by 2020 [1]. While AMD is a complex and multi-factorial disease, the dysfunction and death of retinal pigment epithelium (RPE) cells is believed to play a key role in its disease process [2, 3]. RPE dysfunction in AMD has been attributed to several pathological pathways including the accumulated effects of oxidative stress, toxic metabolites, and inflammation [2]. More recently, endoplasmic reticulum (ER) stress has been suggested as playing an important role in retinal and neural disorders including the atrophic form of AMD [4, 5].

“ER stress” is the accumulation of unfolded or misfolded proteins in the ER lumen that triggers the complex cellular response known as the unfolded protein response (UPR) [5, 6]. This response is widely believed to be mediated through the ER chaperone GRP78, which is normally bound to the luminal domain of three trans-membrane ER proteins: PKR-like endoplasmic reticulum kinase (PERK), Inositol-requiring enzyme 1 (IRE1), and activating transcription factor 6 (ATF6) [5, 7]. During ER stress GRP78 dissociates from these transmembrane proteins to bind the misfolded and unfolded proteins. Loss of GRP78 binding is believed to be a key step in activating the transmembrane proteins and triggering the UPR [8]. Once the UPR is initiated the cell undergoes several adaptive responses including the upregulation of chaperones, including GRP78, decreasing global protein translation, and enhancing ER-associated degradation (ERAD) of misfolded proteins [5, 7]. However, if this adaptive response fails to reestablish ER homeostasis, signaling switches to a proapoptotic pathway [6].

ER stress induced apoptosis occurs through multiple mechanisms. One of the major signal transducers believed to be involved is C/EBP homologous protein (CHOP). Studies have shown that overexpression of CHOP can lead to apoptosis, while CHOP knockout cells attenuated apoptosis in response to ER stress [9–11]. Caspase cascades have also been found to play a key role in ER stress induced apoptosis. The ER membrane localized caspase 4 has been found to be activated specifically by ER stress, and its attenuation by siRNA showed a protective affect specifically against ER stress induced apoptosis [12]. Furthermore caspase 4 has been shown to directly cleave pro-caspase 9 to trigger apoptosis under ER stress [13].

Our laboratory has long been interested in the mechanisms and effects of oxidative and other stresses on human RPE (hRPE) cells. It has been shown that oxidative stress can lead to ER stress, and that over-expression of adaptive mechanisms of the UPR can protect against oxidative stress induced apoptosis [14]. We found that the ER stress signaling inhibitors salubrinal and 4-(2-aminoethyl) benzenesulfonyl fluoride decreased mitochondrial damage and reduced RPE apoptosis induced by ER stress [15]. A growing body of evidence suggests that ER-mitochondrial cross talk plays a significant role in ER stress induced apoptosis through mitochondrial pathways [6, 16, 17]. ER stress releases Ca^2+^ from ER stores and the sustained accumulation of Ca^2+^ in mitochondria leading to proapoptotic mitochondrial alterations including permeability transition, dissipation of the electrochemical potential, relocalization of Bax to mitochondria and the release of cytochrome c and apoptosis-inducing factor from mitochondria [18]. CHOP has also been shown to cause apoptosis through mitochondrial mechanisms by down-regulating Bcl-2 expression and depleting glutathione (GSH) levels [19], and leading to the translocation of Bax from cytosol to mitochondria [20].

Recently, the small mitochondria-encoded neuroprotective peptide humanin (HN) has emerged as a protective factor against a variety of insults including oxidative stress, serum starvation and hypoxia [21] since its initial discovery as a survival factor in the brain of Alzheimer patients [22]. While HN’s exact mechanism of action remains to be fully elucidated, it was shown to have anti-apoptotic effect by binding the pro-apoptotic proteins Bax, tBid and BimEL and blocking cytochrome c release [23–25]. Our laboratory has recently shown that HN protected RPE cells against oxidative stress-induced cell death by enhancing mitochondrial biogenesis and bioenergetics [26] Despite the close interaction between oxidative stress and ER stress [4, 15], the effect of HN on ER stress has not been evaluated in any cell type. In the present study, we investigated the effect of ER stress on mitochondria particularly with reference to its oxidative status, and the effect of HN on alleviating ER stress-induced apoptosis through modulation of mitochondrial GSH.

## Materials and Methods

### Materials

Tunicamycin (TM), brefeldin A (BFA), and thapsigargin (TG) were obtained from Sigma Aldrich (St. Louis, MO). The 24 amino acid Humanin (HN) peptide with the sequence (MAPRGFSCLLLLTSEIDLPVKRRA) was custom synthesized with a purity >98% (Neopeptide, Cambridge, MA).

### RPE Cell culture

The protocol for the preparation and use of cultured fetal human RPE (hRPE) cells was approved by the University of Southern California Institutional Review Board under protocol #HS-947005 (continuing review approved March 6, 2016) and adhered to the tenets of the Declaration of Helsinki. hRPE cells were isolated from human fetal eyes (gestational age 16–18 weeks) obtained from Novogenix Lab (Los Angeles, CA). Primary cultures of hRPE cells were established as described previously [27, 28]. Three donors were used for experiments, and donor to donor variation had a negligible effect on the results. Second to fourth passage cells were used in all experiments. The hRPE cells were initially cultured to 80–90% confluence in Dulbecco’s modified Eagle medium (DMEM, Fisher Scientific, Pittsburgh, PA, USA) with 10% fetal bovine serum (FBS, Gibco BRL, Gaithersburg, MD, USA), 2mM L-glutamine, and 100 μg/mL streptomycin (Sigma, St. Louis, MO); however, the cells achieved confluence by the time of addition of the ER stressors. In order to model the changes found at the edge of the atrophic AMD lesion we utilized confluent non-polarized cells. While the normal RPE monolayer is comprised of an intact layer of polarized cells, at the edge of atrophic AMD lesions the RPE lose their polarization and this change may be associated with their susceptibility to stress and lesion progression [29]. Twelve hours prior to treatment, cells were switched to 1% FBS media. To investigate the apoptotic effect of each ER stressor in a dose dependent manner, cells cultured to confluency on 6-well plates (VWR, Radnor, PA) were treated with TM (1–20 μg/mL), BFA (10–40 μM) or TG (0.5–3 μM). To study the dose dependent effect of HN, cells were pre-treated with HN (1–20 μg/mL) for 12 hours, followed by TM (10 μg/mL) or BFA (40 μM) or TG (3 μM) treatment in the continued presence of HN (10 μg/mL) for an additional 12 hours.

### U-251 Glioma Cell culture

Human glioma cell line U-251 was obtained from Dr. Florence M Hofman (Department of Pathology, University of Southern California, Los Angeles, CA, United States of America). The U-251 cells were cultured in 6-well plates (VWR) to 80–90% confluence in DMEM with 10% FBS, 2 mM L-glutamine, and streptomycin (100 μg/mL). Twelve hours prior to treatment, cells were switched to a culture medium containing 1% FBS. To study the effect of HN, cells were pre-treated with HN (10 μg/mL) for 12 hours, followed by TM (10μg/mL) treatment in the continued presence of HN (10μg/mL) for an additional 12 hours.

### Real-time PCR analysis

Total RNA was isolated from hRPE cells cultured in 6-well plates (VWR) using a RNeasy Mini Kit, as per manufacturer’s protocol (Qiagen, Valencia, CA), and quantified with a spectrophotometer. First strand cDNA synthesis by reverse transcription was achieved with oligo(dT) primer and 1 μg total RNA in a 20 μL reaction volume, as per the manufacturer’s protocol (ImProm-II Reverse Transcription System, Promega, Madison, WI). RT-PCR was performed using SYBR Green Master Mix) with LightCycler 480 (Roche, IN). Quantification analysis of target genes were normalized using GAPDH was used as the internal control. The sequence of primers used were γ-glutamylcysteine synthetase (GCS) forward: 5’- CAG TTG GCT ACT ATC TGT C-3’ Reverse: 5’- GTC TAT TGA GTC ATA TCG GG-3’, GAPDH forward: 5’- GAGTCAACGGATTTGGTCGT-3’ Reverse: 5’- CTTGATTTTGGAGGGATCTCGC-3’ (Valuegene Inc, San Diego, CA). The primer sequences for the other antioxidant genes used in the study are listed in S1 Table. Relative multiples of change in mRNA expression was determined by calculating the ^ΔΔ^C_T_ values.

### Western Blot Analysis

Cells grown to confluence on 6-well culture plates (VWR) were harvested after the specified treatment period and washed with PBS. Protein was extracted from the cells using mammalian protein extraction reagent with protease inhibitor cocktail (Pierce Biotechnology, Rockford, IL), and quantified with a protein assay (Bio-Rad, Hercules, CA). Equal amounts of protein were resolved on 8–16% Tris-HCL polyacrylamide gels (Pierce Biotechnology, Rockford, IL) and transferred to PVDF blotting membranes (Millipore, Billerica, MA). Membranes were probed with rabbit polyclonal anti-gamma glutamyl cysteine ligase, catalytic subunit (GCLC) (Abcam, Cambridge, MA), rabbit anti-cleaved caspase 3 (Cell Signaling Technology, MA), mouse anti-CHOP (Pierce Biotechnology, IL), rabbit anti-GRP78 (Sigma Aldrich, St. Louis, MO), rabbit anti-COX IV (Cell Signaling Technology, MA), rabbit polyclonal anti-GRX2 (GeneTex, Irvine, CA) and rabbit anti-β-Tubulin (Cell Signaling Technology, MA) overnight at 4°C. After incubation with corresponding secondary antibody tagged with horseradish peroxidase, signals were detected using an ECL chemiluminescence system (Pierce Biotechnology, IL). Membranes were then stripped and reprobed with monoclonal anti-GAPDH (Millipore, Billerica, MA). Protein band intensity was measured by Image Studio Software (Li-Cor, Lincoln, NE) [30].

### Apoptosis Assay

DNA cleavage of hRPE cells and U-251 glioma cells grown on Falcon 4-well chamber slides (Corning Inc, Corning, NY) was measured by TdT-mediated dUTP nick-end labeling (TUNEL; In-Situ Cell Death Detection Kit, Roche). Adherent cells from control and treated cells were processed according to the manufacturer’s protocol and cells were analyzed via confocal microscopy (LSM510, Carl Zeiss, Thornwood, NY) and fluorescence microscopy, (Keyence, Itasca, IL) with a DAPI counterstain (Vector Laboratories, Burlingame, CA).

### Confocal Microscopy

hRPE cells were grown on Falcon 4-well chamber slides (Corning Inc.) and treated as described above. After treatment, cells were fixed with 4% paraformaldehyde for 15 minutes. Cells were then blocked and permeabilized with 5% goat serum (Invitrogen, Carlsbad, CA) and 0.3% Triton X-100 for 1 hour. Primary antibodies reactive against activated caspase 3 and caspase 4 (Abcam, Cambridge, MA) were added for overnight prior to addition of FITC-conjugated secondary antibody (Invitrogen, Carlsbad, CA) for 1 hour. Slides were mounted with mounting medium containing DAPI (Vector Laboratories, Burlingame, CA). Slides were examined using a confocal microscope (LSM510, Carl Zeiss, Thornwood, NY).

### Mitosox Assay

Mitochondrial superoxide production was measured by using MitoSOX Red Kit (Life Technologies, Carlsbad, CA) per manufacturer’s protocol. All experiments were performed in CO_2_ incubator at 37°C. Cells were cultured in Falcon 4-well chamber slides (Corning Inc., Corning, NY). For confocal microscopy, Mitosox and ER tracker green (Life Technologies, Carlsbad, CA) was added at 5 μM and 500 μM, respectively, in the medium 30 minutes prior to the end of treatment. Cells were washed with PBS and briefly fixed with 4% paraformaldehyde for 1 minute prior to mounting with mounting medium (Vector Laboratories, Burlingame, CA) and imaging.

### Mitochondrial Fractionation

RPE Cells grown to confluence on T-75 flasks (VWR, PA) were harvested after the specified treatment period and mitochondria were isolated from the cytosolic fraction using a commercial Mitochondria/Cytosol Fractionation Kit (Biovision Inc, Mountain View, CA) as described previously [15]. Cells were homogenized with 40 strokes of a Dounce homogenizer and suspensions were observed under a microscope to ensure proper cell lysis. The final mitochondrial pellet was washed in PBS one more time to ensure purity of mitochondrial fraction [15].

### GSH Analysis

A GSH-Glo glutathione assay (Promega, WI) was used to measure unbound GSH. After isolation of mitochondria as described above, whole mitochondria were used per the manufacturer’s protocol. Processed samples were analyzed using a luminometer and results were expressed as a fraction of control.

A GSH/GSSG-Glo glutathione assay (Promega, WI) was used to measure the ratio of GSH/GSSG in whole cells. Processed samples were analyzed using a luminometer and results were presented as relative ratio of GSH to GSSG in all experimental groups. GSSG levels in mitochondria being extremely low and below the sensitivity of the assay, GSH/GSSG ratios could not be determined in mitochondrial samples.

### Statistical Analysis

Statistical analysis for multiple comparisons was performed using one way ANOVA with Tukey’s or Dunnett’s post hoc test. Student’s t-tests were two-tailed. P<0.05 was considered as significant. The statistic software JMP Pro (Version 11, SAS, Inc., Cary, NC) was used for all statistical analysis.

## Results

**Fig 1:**
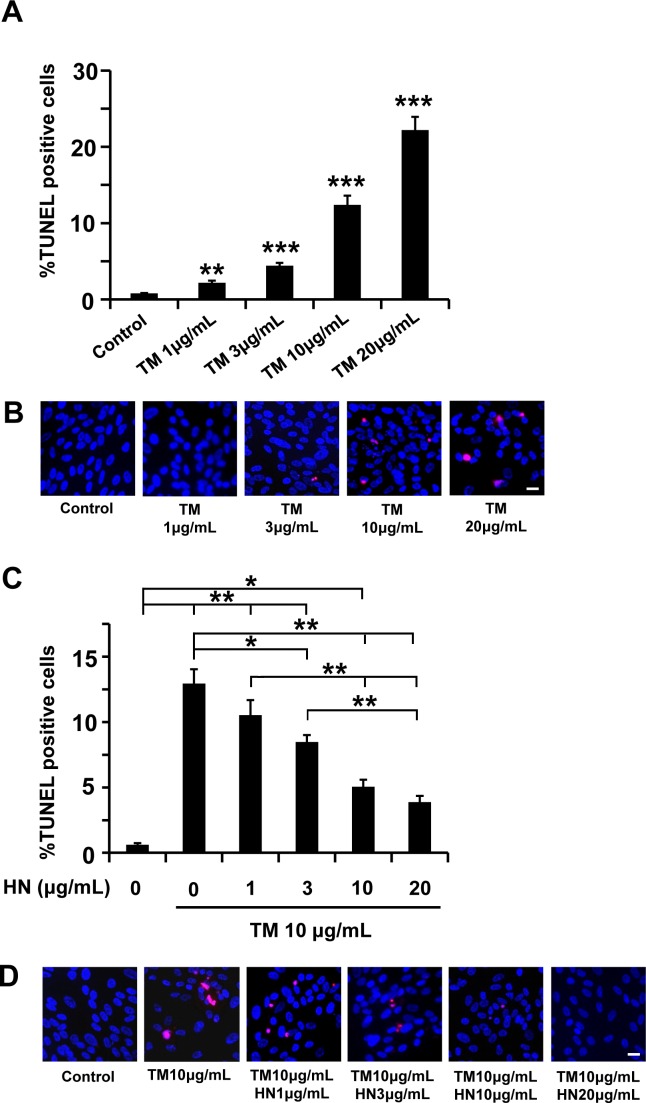
TM-induced apoptosis in RPE cells and protection by HN. Confluent hRPE cells were treated with varying concentrations (1–20 μg/mL) of TM for 12 hours. (**A**) Percentage of TUNEL positive cells increased in a dose-dependent manner with TM treatment. (**B**) Representative images of TUNEL positive cells (red) and nuclei (blue) are shown for each treatment condition. (**C**) Pre-incubation of HN (1–20 μg/mL) for 12 hours protected RPE cells from apoptosis induced by TM (10 μg/mL). (**D**) Representative images are shown for each HN treatment group. Data are mean ± SEM (n = 3). Asterisks represent *p<0.05, **p<0.01, ***p<0.001). Scale bar: 20 μm in B and D.

We examined the effect of three ER stressors; TM (glycosylation inhibitor), BFA (Golgi complex translocation inhibitor), and TG (calcium flux inhibitor) on apoptotic cell death in primary human RPE cells. In our prior studies, we had shown that TM induces ER stress at 3–10 μg/mL for time periods less than 24 hr in hRPE cells [15]. In the current study, we expanded the dose range. TM increased apoptotic cell death in a dose dependent manner in the concentration range of 1–20 μg/mL (Fig 1A and 1B). The percentage of TUNEL positive cells increased significantly (p<0.01) from a mean of 0.8% in control untreated cells to a mean of 2.2% in RPE cells treated with 1 μg/mL TM. The increases in cell death with 3, 10, 20 μg/mL TM treatment were also significantly higher (p<0.001 vs control) and averaged 4.4, 12.4 and 22.2%, respectively (Fig 1A and 1B). We then investigated whether pre-treatment with HN could protect TM treated cells from apoptosis and whether this protection was dose-dependent. Our data demonstrated that, after pre-incubation with HN (1, 3, 10 and 20 μg/mL), TM-induced apoptosis analyzed by TUNEL assay significantly decreased from 13% (no HN treatment) to 10.5% and 8.5% with 1 and 3 μg/mL HN and 5.1% and 3.9% with 10, 20 μg/mL HN (p<0.05 vs TM treated for 3 μg/mL HN and p<0.001 vs TM treated for 10 and 20 μg/mL HN.(Fig 1C and 1D).

**Fig 2:**
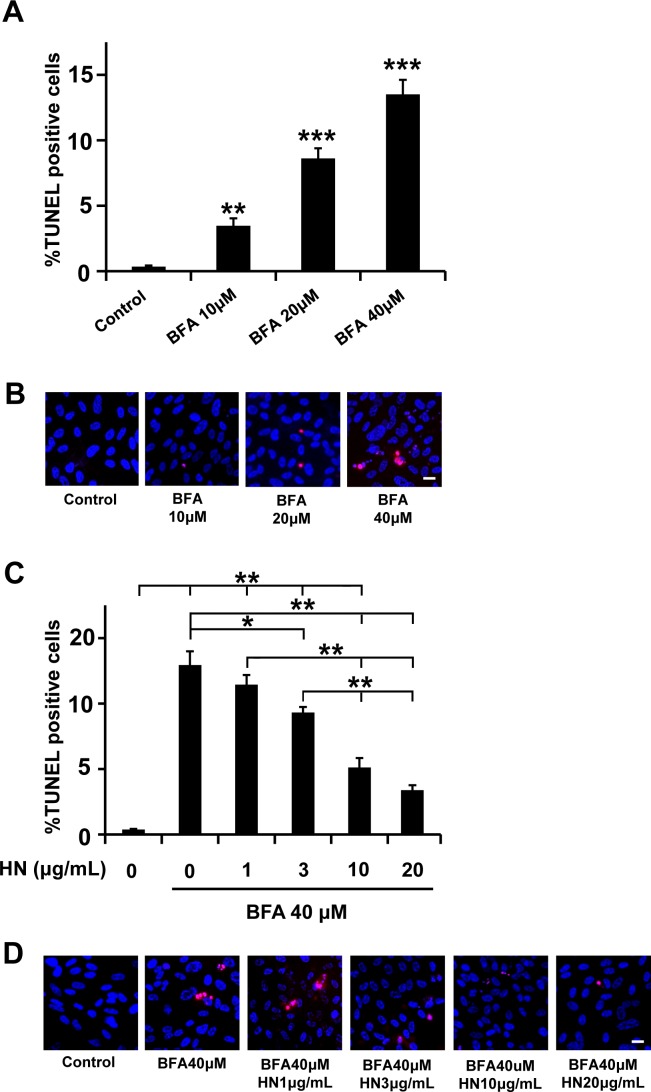
BFA-induced apoptosis in RPE cells and protection by HN. Confluent hRPE cells were treated with varying concentrations (10–40 μM) of BFA for 12 hours. (**A**) Percentage of TUNEL positive cells increased in a dose-dependent manner with BFA treatment. (**B**) Representative images of TUNEL positive cells (red) and nuclei (blue) are shown for each treatment condition. (**C**) Pre-incubation with HN for 12 hours protected RPE cells from apoptosis induced by BFA (40 μM) dose-dependently. (**D**) Representative images are shown for each HN treatment group. Data are mean ± SEM (n = 3). Asterisks represent *p<0.05, **p<0.01, ***p<0.001. Scale bar: 20 μm in B and D.

Similarly, when RPE cells were treated with BFA (10–40 μM) for 12 hr, cell death increased significantly in a concentration-dependent manner (Fig 2A and 2B). The percentage of TUNEL positive cells increased significantly (p<0.01) from a mean of 0.35% in control untreated cells to a mean of 3.5% in RPE cells treated with 10 μM BFA. The increase in cell death with 20 and 30 μM BFA treatment were also significantly higher (p<0.001 vs control) and averaged 8.6%, and 13.5%, respectively (Fig 2A and 2B). We then investigated whether pre-treatment with HN could protect TM treated cells from apoptosis and whether this protection was dose-dependent. Our data demonstrated that, after pre-incubation with HN (1, 3, 10 and 20 μg/mL), BFA-induced apoptosis analyzed by TUNEL assay significantly decreased from 13% (no HN treatment) to 1.4% and 9.3% with 1 and 3 μg/mL HN (p<0.05 vs BFA treated for 3 μg/mL HN and 5.1% and 3.4% with 10 and 20 μg/mL HN (p< 0.01 vs BFA treated; Fig 2C and 2D).

**Fig 3:**
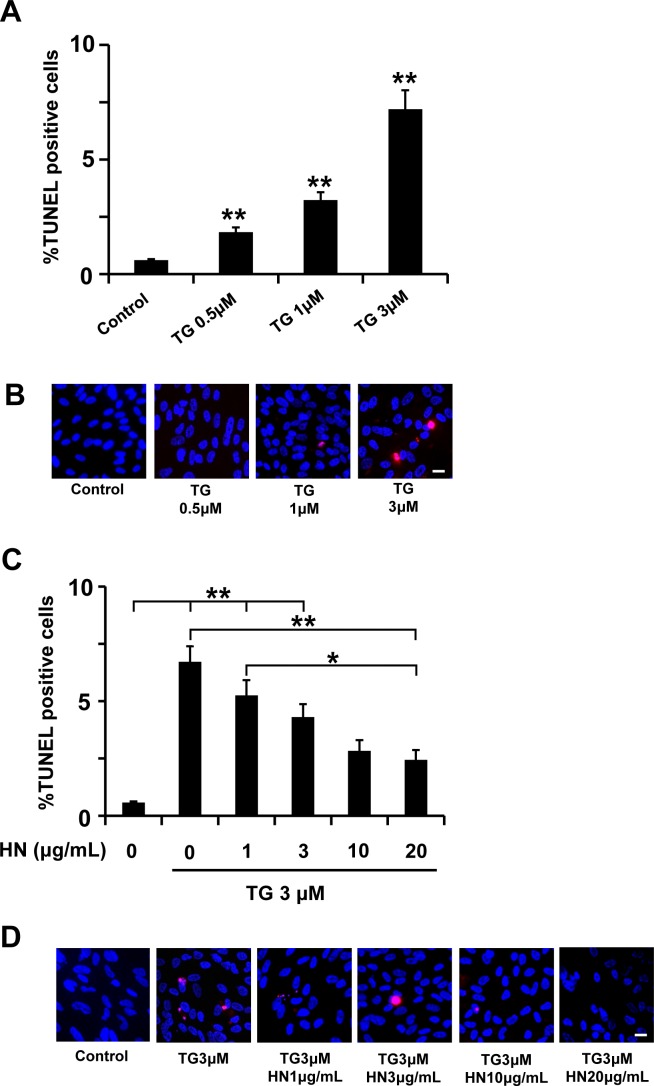
TG-induced apoptosis in RPE cells and protection by HN. Confluent hRPE cells were treated with varying concentrations (0.5–3 μM) of TG for 12 hours. (**A**) Percentage of TUNEL positive cells increased in a dose-dependent manner with TG treatment. (**B**) Representative images of TUNEL positive cells (red) and nuclei (blue) are shown for each experimental condition. (**C**) Pre-incubation with HN for 12 hours protected RPE cells dose-dependently from apoptosis induced by TG (3 μM). (**D**) Representative images are shown for each HN treatment group. Data are mean ± SEM (n = 3). Asterisks represent **p<0.01). Scale bar: 20 μm in B and D.

Fig 3 shows the effect of the TG treatment on RPE apoptosis and HN pre-treatment. TG increased apoptotic cell death in a dose dependent manner in the concentration range of 0.5–3 μM (Fig 3A and 3B). The percentage of TUNEL positive cells increased significantly (p<0.01) from a mean of 0.70% in control untreated cells to a mean of 1.5%, 3.2% and 7.2% at 0.5, 1 and 3 μM TG, respectively (p<0.001 vs control). We then investigated whether pre-treatment with HN could protect TG treated cells from apoptosis and whether the protection was dose dependent. Our data demonstrated that, after pre-incubation with HN (1, 3, 10 and 20 μg/mL), TG-induced apoptosis analyzed by TUNEL assay decreased significantly from 6.7% (no HN treatment) to 5.3%, 4.3%, 2.8% and 2.4% with 1, 3, 10, and 20 μg/mL HN (p<0.01 vs TG-treated, Fig 3C and 3D).

We selected TM as ER stressor in further elucidating the mechanisms of HN protection. TM has been used in several in *vivo* ER induced ocular studies testing potential therapy [31–33].

### TM induced ER stress caused increased GRP78 and CHOP expression

**Fig 4:**
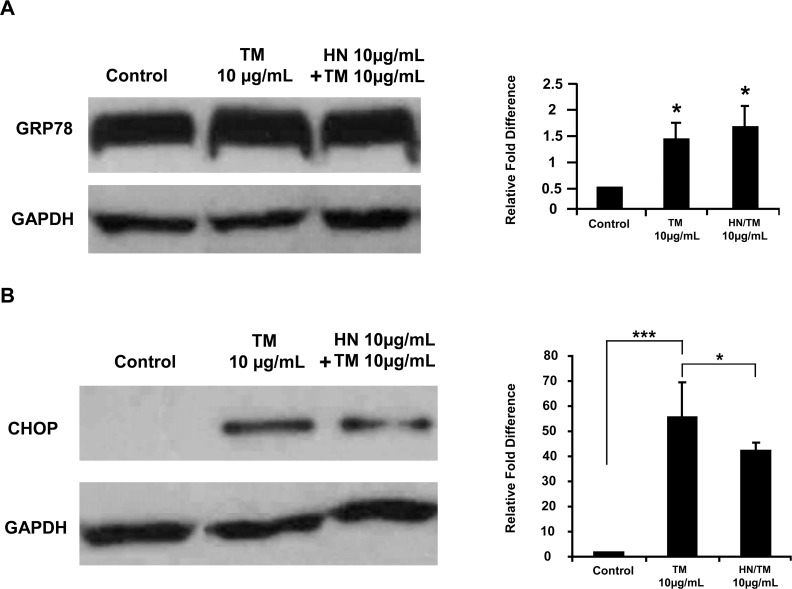
Increased expression of the ER stress markers GRP78 and CHOP by TM treatment of hRPE cells and the effect of HN. Confluent hRPE cells were pretreated for 12 hours with or without 10 μg/mL HN. Cells were then treated with 10 μg/mL HN and/or 10 μg/mL TM for 12 hours. (**A**) Expression of GRP78 by Western blot analysis was significantly higher in TM and HN plus TM groups compared to control. (n = 4, *p < 0.05). (**B**) Expression of CHOP by Western blot analysis was significantly different between TM and HN plus TM groups compared to controls. However, treatment with HN along with TM reduced the expression of CHOP as compared to TM alone. Bar graph represents protein expression quantified by densitometry normalized to GAPDH. Data are mean ± SEM (n = 3). Asterisks represent ***p < 0.001, **p < 0.01. C- Control.

hRPE cells were treated with 10 μg/mL TM for specified time periods of 1, 6, 12, and 24 hours. DMSO (0.1%) was used as a vehicle for the control group. Preliminary studies revealed that the maximal effect was found with 12 hour treatment (data not shown). In all subsequent experiments we induced ER stress with10 μg/mL TM for 12 hours. As shown in Fig 4, protein expression of the ER stress marker proteins GRP78 and CHOP was significantly elevated with TM treatment at 12 hours (p<0.05 and p<0.001 respectively, vs. controls). HN pretreatment overnight followed by co-treatment for 12 hours caused no further significant change in GRP78 expression compared to TM treated cells. TM treatment resulted in a significant increase in CHOP expression as compared to controls (Fig 4B). On the other hand, CHOP was significantly lower in HN co-treated vs. TM treated cells (p<0.05).

### HN protects against ER stress induced apoptosis and attenuates activated caspase 3 and caspase 4

**Fig 5:**
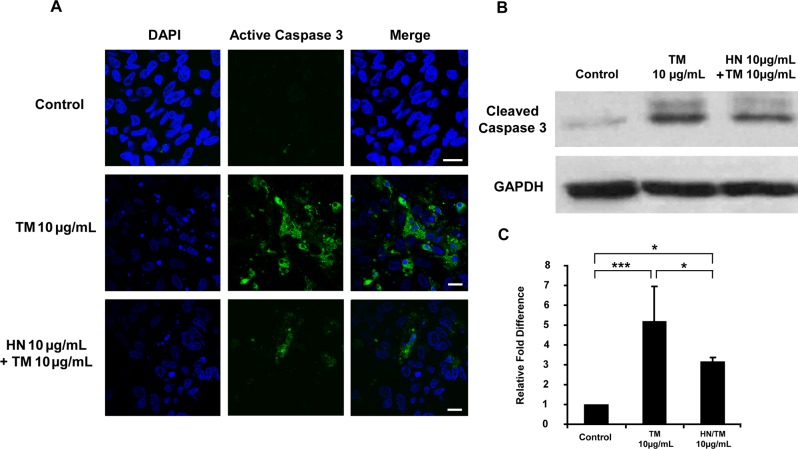
Activation of caspase 3 by TM-induced ER stress in hRPE cells and suppression of activated caspase 3 by HN. Confluent hRPE cells were pretreated for 12 hours with or without 10 μg/mL HN. Cells were then treated with 10 μg/mL HN and/or 10 μg/mL TM for 12 hours. (**A**) Western blot analysis of total cell lysates probed with active caspase 3 antibody showed increased amounts of active caspase 3 with TM, and attenuation of active caspase 3 with HN. (**B**) Protein expression quantified by densitometry as shown as a ratio normalized with GAPDH. Data are mean ± SEM (n = 3). Asterisks represent *p<0.05, ***p<0.001.

**Fig 6:**
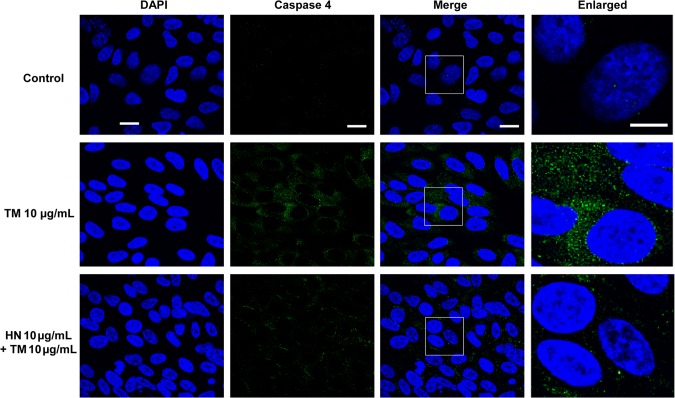
Activation of caspase 4 by TM-induced ER stress in hRPE cells. Confluent hRPE cells were pretreated for 12 hours with or without 10 μg/mL HN. Cells were then treated with 10 μg/mL HN and/or 10 μg/mL TM for 12 hours. Immunostaining with active caspase 4 antibody showed the activation of caspase 4 (green) by confocal microscopy. DAPI (blue) was used to counterstain the nucleus. Scale bar: 10 μm in DAPI, Caspase 4 and Merged panels, and 5 μm in enlarged panels.

As shown in Fig 1, TM exposure resulted in the generation of significant number of apoptotic RPE cells in a dose dependent manner. Our data further revealed that HN co-treatment of hRPE resulted in a marked reduction in the number of apoptotic cells compared to cells treated with TM alone. In addition, we found that HN co-treatment also decreased activated caspase 3 as determined by immunofluorescence and protein expression compared to TM alone (Fig 5A–5C). To explore the effect of HN on ER stress specific apoptotic pathways, we studied the expression of the ER stress specific caspase 4. TM treatment resulted in an increase in immunostaining for caspase-4 as compared to untreated cells which exhibited negligible or no staining. HN co-treatment markedly reduced caspase 4 expression compared to cells treated with TM alone (Fig 6). These results provide supportive evidence that HN has an anti-apoptotic effect against ER stress in hRPE cells.

### HN suppresses ER stress induced mitochondrial superoxide production

**Fig 7:**
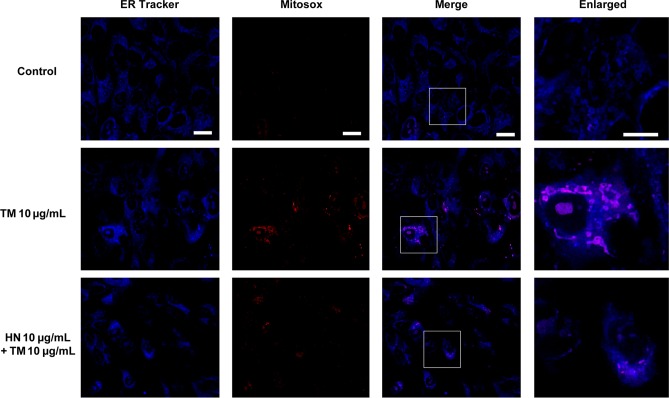
Elevation of mitochondrial superoxide by TM-induced ER stress and decreased mitochondrial superoxide with HN co-treatment. Confluent hRPE cells were pretreated for 12 hours with or without 10 μg/mL HN. Cells were then treated with 10 μg/mL HN and/or 10 μg/mL TM for 12 hours. Increased superoxide in mitochondria is shown by Mitosox labeling (red) against an ER counter-stain (blue). Scale bar: 10 μm in ER Tracker, MitoSox and Merged panels, and 5 μm in enlarged panels.

We wished to study the relationship between ER stress induced apoptosis and its link to mitochondria by measuring mitochondrial reactive oxygen species (ROS) production. To achieve this, we performed Mitosox assay to measure the amount of mitochondrial superoxide using immunoflourescence. In these experiments, TM treated cells clearly showed increased superoxide production compared to control cells. HN co-treated cells showed a pronounced decrease in the immunofluorescence of superoxide compared to cells treated with TM alone (Fig 7).

### ER stress upregulates γ-GCS expression, with no appreciable changes in other major anti-oxidant enzymes

**Fig 8:**
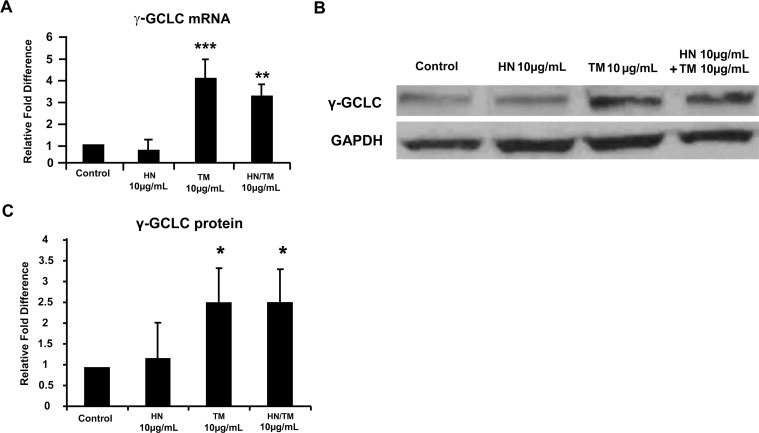
Increased expression of γ-GCLC by TM-induced ER stress in hRPE cells and the effect of HN treatment. Confluent hRPE cells were pretreated for 12 hours with or without 10 μg/mL HN. Cells were then treated with 10 μg/mL HN and/or 10 μg/mL TM for 12 hours. (**A**) RT-PCR analysis of γ-GCLC showed a significant increase in mRNA expression with TM compared to control (n = 4, ***p<0.001, **p<0.01). (B) Western blot analysis of total cell lysates probed with γ-GCLC antibody showed increased expression of γ-GCLC protein with TM compared to control. The γ-GCLC expression remained elevated with HN co-treatment. (**B**) Figure shows a representative western blot from protein expression experiment in the presence and absence of TM and HN. (C) Bar graph showing γ-GCLC protein expression quantified by densitometry as shown as a ratio normalized to GAPDH. Data are mean ± SEM (n = 3). Asterisks represent *p<0.05

We next studied the expression of several anti-oxidant enzymes in relation to ER stress and HN. These studies were performed with both TM and HN at 10 μg/mL. We determined both mRNA and protein expression of the anti-oxidant enzymes catalase, GRX-1, GRX-2, TRX-1 and SOD-II (S1 Table). No significant changes were apparent in the gene or protein expression all of the above enzymes either with TM treatment or cotreatment with HN as compared to untreated cells (S1 and S2 Figs) except for GRX-2 for which the gene expression was increased with TM and TM + HN groups while there was no change at the protein level as compared to controls (S3 Fig). Since GRX-2 is a glutaredoxin localized in mitochondria, we further confirmed that GRX-2 protein expression did not change significantly among control, TM-treated and TM+HN treated groups when mitochondrial preparations from these groups were analyzed (S3 Fig). However, TM exposure caused a significant mRNA and protein elevation in γ-GCLC, the rate limiting enzyme of GSH biosynthesis (p<0.001, p<0.05; Fig 8). HN co-treatment did not result in any further increase in GCLC protein expression (Fig 8).

### HN restores mitochondrial GSH to control levels after ER stress

**Fig 9:**
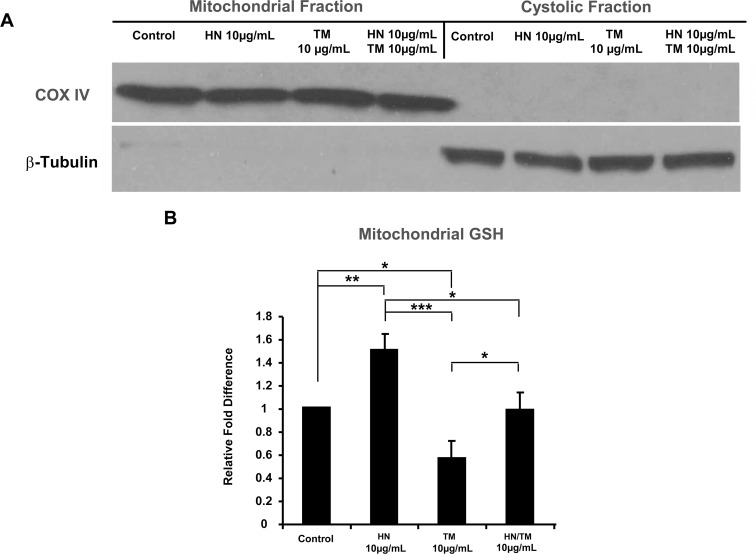
HN restores mitochondrial GSH from depleted levels due to ER stress. Confluent hRPE cells were pretreated for 12 hours with or without 10 μg/mL HN. Cells were then treated with 10 μg/mL HN and/or 10 μg/mL TM for 12 hours. Cells were then fractionated into mitochondrial and cytosolic components using a mitochondrial/cytosol fractionation kit. (A) Western blots of COX IV (mitochondrial marker) and β-Tubulin (cytosolic marker) revealed there is negligible cross-contamination between the two fractions. (B) Mitochondrial GSH measurements showed decreased mitochondrial GSH with TM treatment, and repleted mitochondrial GSH with HN co-treatment. Data presented in the bar graph are mean +/- SD of 3 experiments. (*p<0.05, **p<0.01, ***p<0.001)

Given the important role of mitochondria in the oxidative and apoptotic status of the cell and to further elucidate the phenomenon of ER-mitochondrial cross-talk, we determined the levels of GSH in the whole cell lysate and in mitochondrial compartment of hRPE cells. Cellular GSH decreased significantly with TM treatment as compared to untreated controls as we had reported earlier [15]. (S4 Fig). Cotreatment with HN showed a trend for an increase in cellular GSH vs TM group but it did not reach statistical significance. The GSH/GSSG ratio was significantly reduced with TM and HN cotreatment caused a significant increase in the GSH/GSSG ratio (S4B Fig). HN treatment alone did not alter either the GSH levels or GSH/GSSG ratio in RPE cells. Using a mitochondrial-cytosol fractionation kit, we obtained pure enriched fractions of mitochondria from treated cells (Fig 9A). Analysis of GSH in the mitochondrial fractions revealed a significant decrease in GSH with TM treatment compared to control (p<0.05) (Fig 9B). HN co-treatment was found to significantly increase GSH levels compared to TM treatment alone (p<0.05) to a level similar to that of the control. Of note, we also found a significant elevation in GSH levels in HN treatment alone compared to the control (p<0.01, Fig 9B).

### Effect of TM on glioma cell line U-251

To understand whether or not the protective effect of HN in ER-stress was more general, we performed additional studies using the U-251 glioma cell line. TUNEL staining revealed TM induced apoptosis in a dose-dependent manner in U-251 cells (S5A and S5B Fig). The percentage of TUNEL positive cells increased significantly (p<0.01 for 1 and 3μg/mL TM and p<0.001 for 10 and 20 μg/mLTM). We then investigated whether pre-treatment with HN could protect TM treated cells from apoptosis and whether the protection was dose dependent. Our data demonstrated that, after pre-incubation with HN (1, 3, 10 and 20 μg/mL), TM-induced apoptosis analyzed by TUNEL assay decreased from 13.9% (no HN treatment) to 13.2%, 11.2%, 7.4% and 5.1% with 1, 3, 10, and 20 μg/mL HN, respectively. The decreases with 10 and 20 μg/mL HN pre-treatment were significantly different from TM treated RPE cells (p<0.01, S5C and S5D Fig).

## Discussion

In this study, we show that multiple ER stressors cause apoptotic cell death in primary human RPE cells in a dose-dependent manner. TM induced ER stress in hRPE cells was characterized by an elevation of the ER stress markers GRP78 and CHOP. TM exposure resulted in apoptotic cell death of RPE and increases in activated caspase 3 and caspase 4. ER stress increased mitochondrial superoxide production and caused a significant decline in mitochondrial GSH. Further, we show that co-treatment with the mitochondria-derived peptide HN offered dose-dependent protection against ER stress induced apoptosis. This beneficial effect was achieved in part by the restoration of mitochondrial GSH in HN co-treated hRPE cells.

In our previous work, we reported the effect of several known stimuli of ER stress in human RPE cells and have further elucidated the mechanism of inhibition of ER-induced cell death by αB crystallin (15). For the present study, we chose the ER stressor TM because of its reported use in *in vivo* ocular studies [31–33]. TM treated hRPE cells demonstrated a robust ER stress response with the upregulation of the common ER stress markers GRP78 and CHOP. CHOP is a key signal transducer in the UPR and is considered one of the pro-apoptotic mediators in ER stress [9–11]. We had shown earlier that with over-expression of the cytoprotective heat shock protein αB-crystallin, CHOP elevation in ER stress can be suppressed leading to an anti-apoptotic response [15]. Interestingly, in our current study HN co-treatment showed a significant decrease in CHOP elevation with TM suggesting that HN may play an anti-apoptotic role in ER stress through modulation of the balance between adaptive functions and apoptosis in the UPR.

Limited data are available for the physiological plasma levels of HN in humans. Normal HN levels averaged 124 pg/mL and increased to 205 pg/mL in an impaired glucose group of patients [34]. Members of our group (P.C.) found a somewhat higher range (762–913 pg/mL) in healthy adults and an increase in patients with type 1 diabetes mellitus [35]. In another study, Widmer et al. [36] reported higher plasma HN levels (2.2 ±1.5 ng/mL) in patients with normal coronary endothelial function which decreased to 1.3 ±1.1 ng/mL with cardiac dysfunction. Information on cellular and tissue levels of HN is scarce. In studies on pharmacokinetics of HN in rodents, the tissue levels of HN were found to be lower than plasma levels in the liver and brain and heart [37]. Our recent work in human RPE monolayers *in vitro* showed that cellular HN levels averaged 500 pg/mg protein in non-polarized RPE and increased by 3-fold when they are polarized [27]. The concentration of HN in microanatomic domains of the outer retina such as the subretinal space, the apical RPE cell surface, or interphotoreceptor matrix remains to be determined.

The protective effect of exogenous HN and its analogs has been reported for several cell tissues and cell types that includes cardiac myoblasts and endothelial cells [23, 36, 38, 39], cortical neurons [40] human lymphocytes [41], Cos-7 and colon cancer cells [24], and pancreatic β-cells [42]. The cellular protection in all the above studies was achieved using exogenous administration of HN or its analogs in the micromolar range. There has been no rationale provided for the need for much higher concentrations of exogenous HN peptide for cellular effects compared to physiological endogenous serum concentrations. Possibilities include differences in pharmacodynamics, efficiency of uptake by target cells, roles of potential additional binding partners or concentrations in microdomains when comparing exogenous vs endogenous peptide. Accordingly, we reported that HN (10 μg/mL; 3.8 μM) caused a significant attenuation of oxidant induced apoptosis in RPE cells [27]. Thus, the concentration of HN used to study ER-stress induced cell death in RPE in this study is in the same range as what is generally used in the literature for many different cell types.

ER stress induced apoptosis is known to occur through several different mechanisms. One mechanism is through the ER localizing human caspase 4, which has been found to be activated specifically by ER stress [12]. Caspase 4 possesses a caspase recruitment domain (CARD), and has been shown to be able to directly cleave pro-caspase 9 to trigger apoptosis [13]. A dual role for caspase 4 was suggested in inflammation and ER stress induced apoptotic response in hRPE [43]. Our results confirm caspase 4 activation with ER stress in hRPE cells. The decrease in caspase 4 with HN co-treatment further supports a role of ER stress specific protection by HN.

Another major mechanism of ER stress induced apoptosis is believed to occur through the abundant cross-talk between the ER and mitochondria. The mitochondria is well known to be a key player in both the apoptotic and oxidative state of a cell, and it is becoming increasingly clear that the ER works in close conjunction with the mitochondria in many of these processes [6, 16–18]. CHOP has been shown to lead to decreased Bcl-2 expression, translocation of Bax to the mitochondria, and depleted GSH levels [21]. The process of ER associated degradation depletes reducing equivalents [4], and protein folding itself has been found to generate ROS through ERO1; indeed it has been suggested that protein folding could account for approximately 25% of a cell’s ROS production [44]. Thus, the ER presents itself as a potent source of oxidative stress that can contribute to apoptosis through ROS sensitive mitochondrial pathways. As evidence for this crosstalk with this link between ER and mitochondria, we found that ER stress caused an increase in mitochondrial superoxide in the present study. Our study also found a significant elevation of active caspase 3, a common downstream mediator of mitochondrial apoptotic pathways.

To further explore links between ER stress and mitochondria we assessed for the protein and mRNA expression pattern of several anti-oxidant enzymes. We found no significant changes in cytosolic enzymes catalase, TRX-1, GRX-1 and the mitochondrial superoxide dismutase (SOD-II) with TM or HN pre-treatment. It is of interest that, similar to lack of change in expression of SOD-II with ER, our earlier study found a similar result when H2O2 was used as a RPE stressor [45]. However, the mitochondria associated glutaredoxin (GRX-2) showed gene upregulation with both TM and HN while GRX-2 protein was not altered with either TM as well as TM+HN co-treatment. The lack of change in GRX-2 protein was also found to be true for isolated mitochondrial fractions. This phenomenon of transcriptional regulation of GRX-2 with no apparent translational alterations cannot be explained at the present time and needs further study. A significant increase in the mRNA and protein expression of γ-GCLC, the rate limiting enzyme in GSH production, with TM treatment was observed. This may occur by the mechanism of PERK activation of Nrf2, which is reported to be an important inducer of γ-GCS expression [46–48].

GSH is a critical tripeptide thiol known to play a variety of important roles in the cell including cell signaling. It is perhaps most well known for being a critical anti-oxidant, and has arisen as the main line of defense in maintaining the proper oxidant state of mitochondria [49,50]. It is produced exclusively in the cytosol, but distributes into multiple organelles including the ER and mitochondria. GSH depletion has been shown to predispose cells to apoptosis by increased mitochondrial membrane permeability and caspase 3 activation [51]. Our laboratory has previously shown that ER stress leads to mitochondrial GSH depletion in RPE [15], and this was confirmed in the present study. Further we show that, HN co-treatment lead to a restoration of GSH levels and decreased mitochondrial superoxide compared to TM treatment alone.

While HN has been shown to protect cells against oxidative stress [27], the exact underlying mechanism of action is not clearly understood. Our laboratory has recently found that exogenous administration of HN leads to increased HN levels within hRPE [27]. One possible mechanism of action is that HN has been shown to be able to act intracellularly by binding the pro-apoptotic proteins Bax, tBid and BimEL thereby blocking mitochondrial apoptotic pathways [24–27]. Our previous study also provided evidence that HN increased mitochondrial biogenesis in RPE cells [27]. Restoration of mitochondrial GSH presents another mode of action through which HN could protect against apoptosis.

The homeostasis and steady state level of GSH in a cell is governed by its biosynthesis, utilization and release. We observed a significant increase in γ-GCS with TM treatment that would suggest that the biosynthetic pathway was upregulated to compensate for TM induced depletion of cellular GSH levels. It is of interest that we previously showed that oxidative stress causes increased GSH efflux from hRPE cells, and this process was mediated by the multi-drug resistance protein (MRP)-1 [52]. On the other hand, we found that HN significantly elevated the levels of mitochondrial GSH pool from the reduced levels caused by ER stress. Since mitochondria do not possess GSH synthetic machinery, one possible mechanism through which mitochondrial GSH can be elevated is by its import from the cytosol. Co-treatment with HN did not further change total cellular GSH. The mitochondrial dicarboxylate and oxoglutarate carrier proteins have been shown to import GSH [48], and it is likely that these proteins contribute to GSH in hRPE mitochondria. We hypothesize that future studies could reveal functional or quantitative changes in these GSH carrier proteins with HN treatment.

It is well established that a primary site of AMD pathology is the RPE [53, 54]. The importance of oxidative stress in the pathogenesis and progression of AMD has also been well documented [55, 56]. AMD is characterized by the accumulation of lipofuscin and extracellular deposits (drusen), and the retina and RPE are chronically exposed to oxidative stress through intense light exposure, high oxygen consumption and metabolic activity, and high levels of unsaturated fatty acids. Oxidative stress can initiate the onset of ER stress and we have recently shown that there is a close link between ER stress and mitochondrial dysfunction in human RPE cells [15, 16, 57]. Indeed, the role of ER stress in AMD pathogenesis has been reported in experimental animal models of AMD [4,58,59]. Further, one major impact of oxidative stress is the initiation of cellular senescence, and premature senescence has been suggested as a potentially important pathophysiological mediator of RPE degeneration [60–62].

In conclusion, our study demonstrates that in hRPE, ER stress induces significant ER-mitochondrial cross-talk with several apoptotic pathways activated including mitochondrial caspase 3 and ER stress specific caspase 4. Our study shows that ER stress induces significant mitochondrial oxidative stress on hRPE through increased mitochondrial superoxide and depleted mitochondrial GSH. Further we demonstrate that HN exhibits an anti-apoptotic effect against ER stress in hRPE, and significantly restores depleted mitochondrial GSH in ER stressed hRPE. Our previous work showed that HN protects RPE from oxidative stress induced apoptosis and oxidative stress induced senescence [15]. Here we show that HN is also protective against ER stress and thus should be investigated further as a potential candidate for therapy for diseases such as AMD that involve both oxidative and ER stress.

## Supporting Information

S1 Fig Unchanged mRNA expression of anti-oxidant enzymes by TM-induced ER stress in hRPE cells. Confluent hRPE cells were pretreated for 12 hours with or without 10 μg/mL HN. Cells were then treated with 10 μg/mL HN and/or 10 μg/mL TM for 12 hours. RT-PCR analysis of the anti-oxidant enzymes catalase, GRX-1, TRX-1, and SOD-II showed no change in mRNA expression with TM compared to control. Data are mean ± SEM (n = 3).
(TIF)

S2 Fig Unchanged protein expression of anti-oxidant enzymes by TM-induced ER stress in hRPE cells. Confluent hRPE cells were pretreated for 12 hours with or without 10 μg/mL HN. Cells were then treated with 10 μg/mL HN and/or 10 μg/mL TM for 12 hours. RT-PCR analysis of the anti-oxidant enzymes catalase, GRX-1, and TRX-1 show no change in protein expression with TM compared to control. Data are mean ± SEM (n = 3).
(TIF)

S3 Fig Expression changes in GRX-2 in TM-induced ER stress in hRPE cells and the effect of HN treatment. Confluent hRPE cells were pretreated for 12 hours with or without 10 μg/mL HN. Cells were then treated with 10 μg/mL HN and/or 10 μg/mL TM for 12 hours. (A) RT-PCR analysis of GRX-2 showed a significant increase in mRNA expression with TM and HN plus TM groups compared to control (n = 3, **p<0.01, *p<0.05). (B,C) Western blot analysis of total cell lysates probed with GRX-2 antibody showed no significant changes in GRX-2 protein expression with TM or HN compared to control. (B) Figure shows a representative Western blot from protein expression in whole cell lysate. (C) Bar graph showing GRX-2 protein expression quantified by densitometry as shown as a ratio normalized to GAPDH. (*p<0.05). (D). Western blot analysis of mitochondrial fractions probed with GRX-2 antibody showed no significant changes in the GRX-2 protein expression in TM or TM+HN compared to untreated control. (E). Densitometry analysis of the blots from three independent experiment normalized to pyruvate dehydrogenase (PDH) is shown. Data are mean ± SEM (n = 3).
(TIF)

S4 Fig Cellular GSH and GSH/GSSG ratios in hRPE cells. Confluent hRPE cells were pretreated for 12 hours with or without 10 μg/ml HN. Cells were then treated with 10 μg/ml TM for 12 hours. (A). Cellular GSH levels showed a decrease with TM treatment. (B) The GSH/GSSG ratio decreased significantly with TM treatment and showed an increase with HN+TM cotreatment. Data are mean ± SEM (n = 3). Asterisks represent *p<0.05, **p<0.01.
(TIF)

S5 Fig TM induced apoptosis in U-251 glioma cells and protection by HN. Confluent U-251 cells were treated with TM for 12 hours. (A) Percentage of TUNEL positive cells increased in a dose-dependent manner with TM treatment. (B) Representative images of TUNEL positive cells (red) and nuclei (blue) are shown per each treatment condition. (C) Pre-incubation with HN for 12 hours protected TM-induced apoptosis with TM (10 μg/mL) dose-dependently. (D) Representative images are shown for each group. Data are mean ± SEM (n = 3). Asterisks represent **p<0.01, ***p<0.001. Scale bar: 20 μm in B and D.
(TIF)

S1 Table Primer sequences and antibodies for Catalase, GRX-1, GRX-2, TRX-1 and SOD-II. (PDF)
