# Supplementary figures and images for: Humanin Protects RPE Cells from Endoplasmic Reticulum Stress-Induced Apoptosis by Upregulation of Mitochondrial Glutathione
Source: PLoS One. 2016 Oct 26;11(10):e0165150. doi: 10.1371/journal.pone.0165150 (PMC5081188; doi:10.1371/journal.pone.0165150)

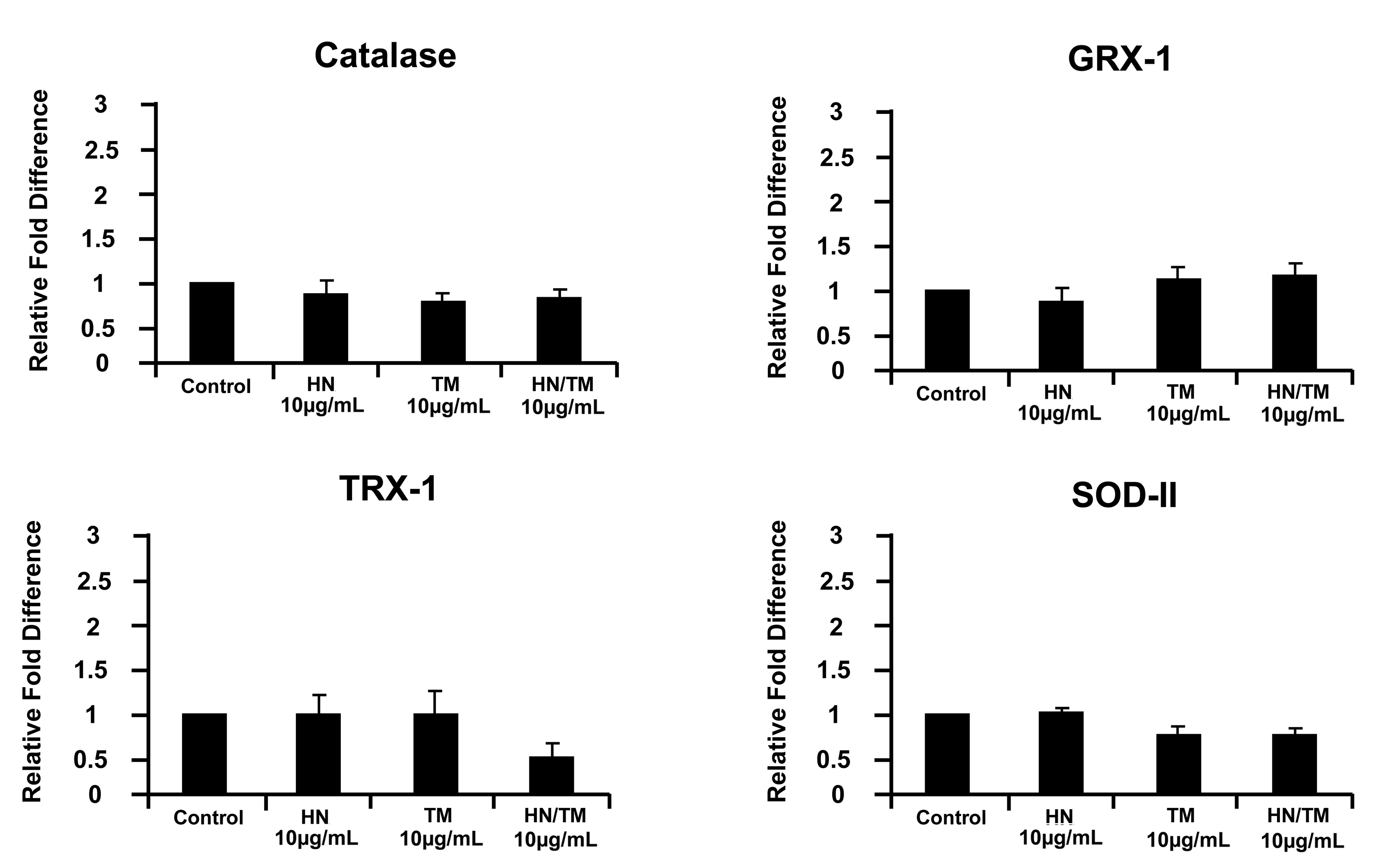

Supplement: S1 Fig — Confluent hRPE cells were pretreated for 12 hours with or without 10 μg/mL HN. Cells were then treated with 10 μg/mL HN and/or 10 μg/mL TM for 12 hours. RT-PCR analysis of the anti-oxidant enzymes catalase, GRX-1, TRX-1, and SOD-II showed no change in mRNA expression with TM compared to control. Data are mean ± SEM (n = 3). (TIF) [file pone.0165150.s001.tif]

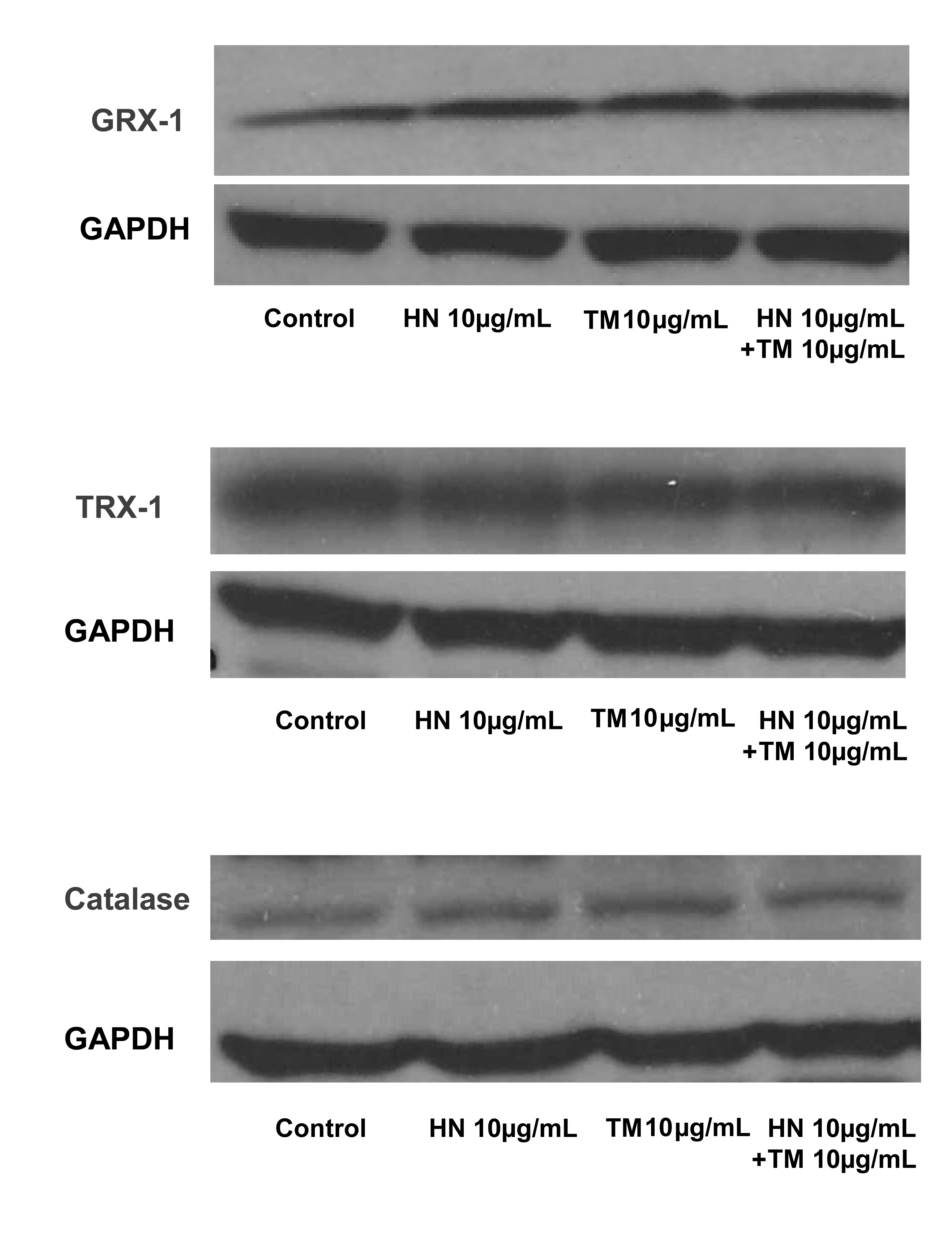

Supplement: S2 Fig — Confluent hRPE cells were pretreated for 12 hours with or without 10 μg/mL HN. Cells were then treated with 10 μg/mL HN and/or 10 μg/mL TM for 12 hours. RT-PCR analysis of the anti-oxidant enzymes catalase, GRX-1, and TRX-1 show no change in protein expression with TM compared to control. Data are mean ± SEM (n = 3). (TIF) [file pone.0165150.s002.tif]

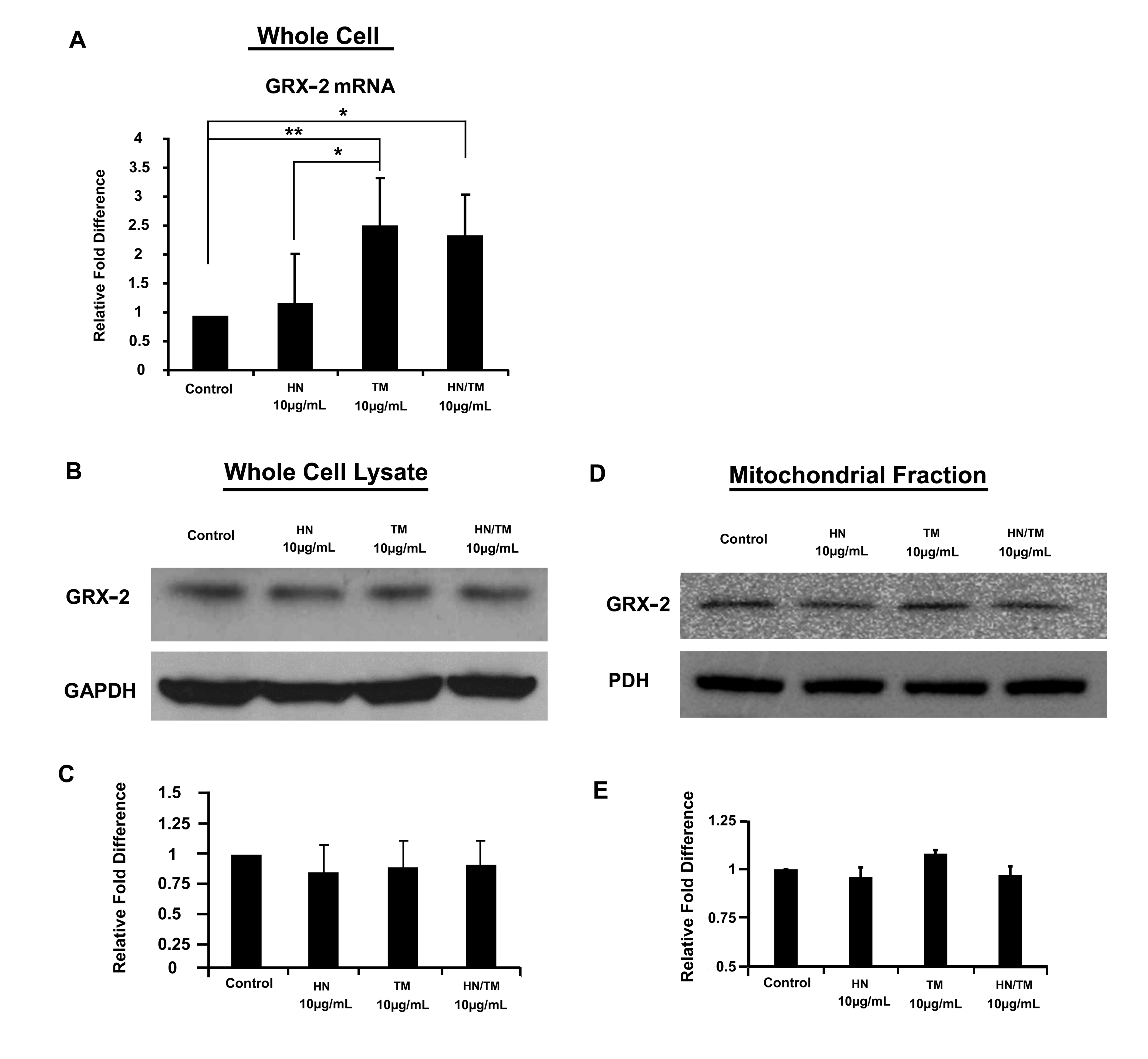

Supplement: S3 Fig — Confluent hRPE cells were pretreated for 12 hours with or without 10 μg/mL HN. Cells were then treated with 10 μg/mL HN and/or 10 μg/mL TM for 12 hours. (A) RT-PCR analysis of GRX-2 showed a significant increase in mRNA expression with TM and HN plus TM groups compared to control (n = 3, **p<0.01, *p<0.05). (B,C) Western blot analysis of total cell lysates probed with GRX-2 antibody showed no significant changes in GRX-2 protein expression with TM or HN compared to control. (B) Figure shows a representative Western blot from protein expression in whole cell lysate. (C) Bar graph showing GRX-2 protein expression quantified by densitometry as shown as a ratio normalized to GAPDH. (*p<0.05). (D). Western blot analysis of mitochondrial fractions probed with GRX-2 antibody showed no significant changes in the GRX-2 protein expression in TM or TM+HN compared to untreated control. (E). Densitometry analysis of the blots from three independent experiment normalized to pyruvate dehydrogenase (PDH) is shown. Data are mean ± SEM (n = 3). (TIF) [file pone.0165150.s003.tif]

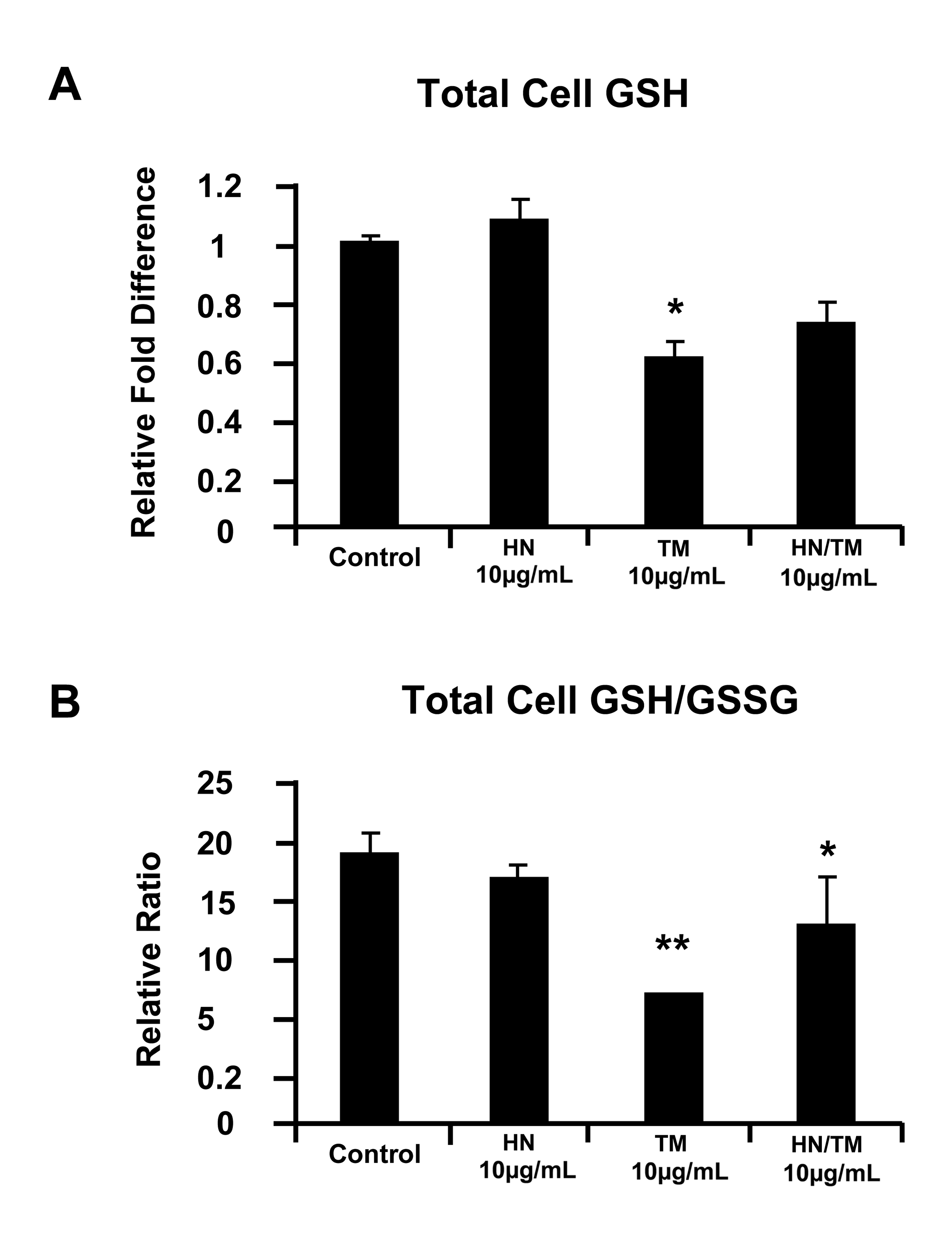

Supplement: S4 Fig — Confluent hRPE cells were pretreated for 12 hours with or without 10 μg/ml HN. Cells were then treated with 10 μg/ml TM for 12 hours. (A). Cellular GSH levels showed a decrease with TM treatment. (B) The GSH/GSSG ratio decreased significantly with TM treatment and showed an increase with HN+TM cotreatment. Data are mean ± SEM (n = 3). Asterisks represent *p<0.05, **p<0.01. (TIF) [file pone.0165150.s004.tif]

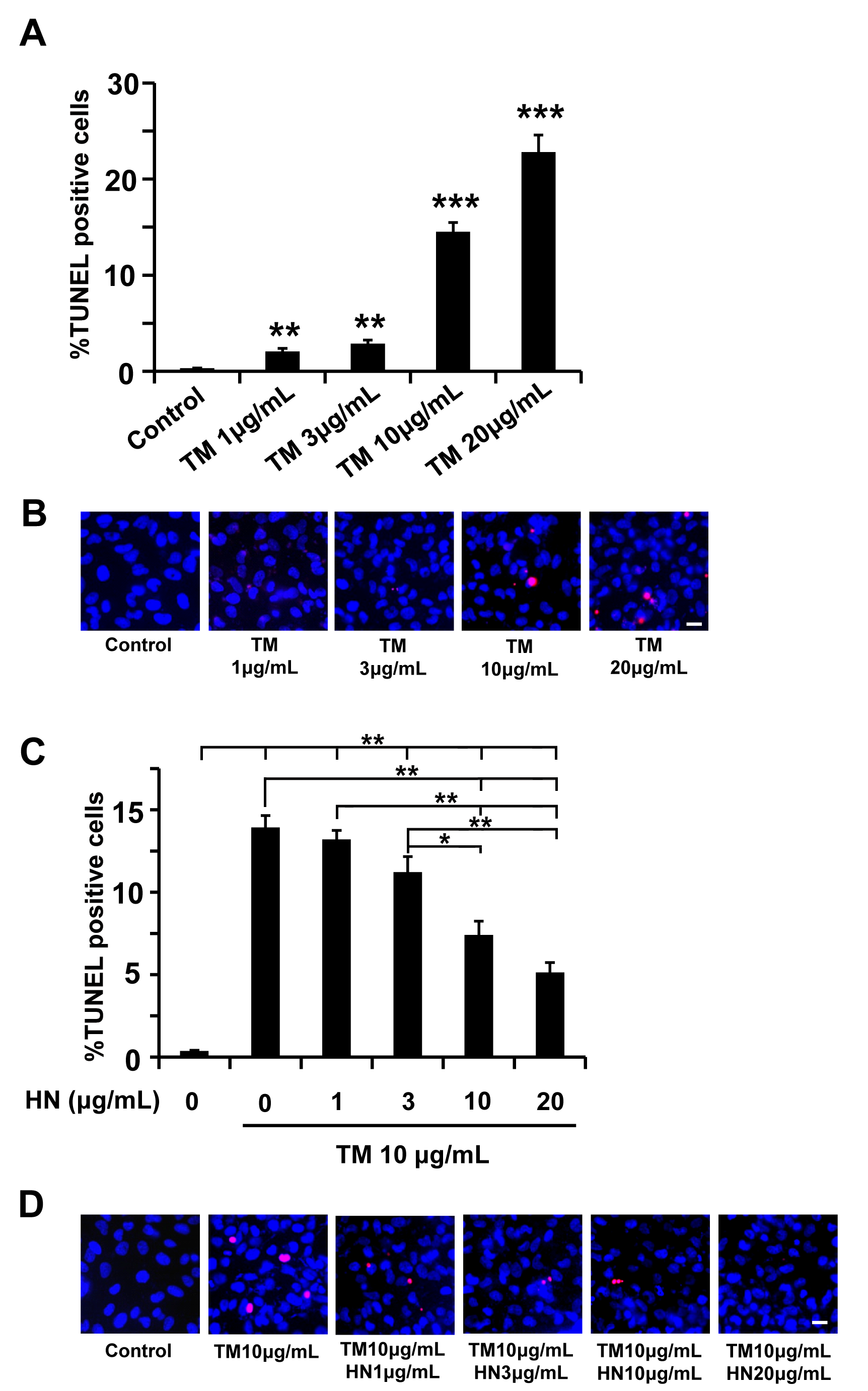

Supplement: S5 Fig — Confluent U-251 cells were treated with TM for 12 hours. (A) Percentage of TUNEL positive cells increased in a dose-dependent manner with TM treatment. (B) Representative images of TUNEL positive cells (red) and nuclei (blue) are shown per each treatment condition. (C) Pre-incubation with HN for 12 hours protected TM-induced apoptosis with TM (10 μg/mL) dose-dependently. (D) Representative images are shown for each group. Data are mean ± SEM (n = 3). Asterisks represent **p<0.01, ***p<0.001. Scale bar: 20 μm in B and D. (TIF) [file pone.0165150.s005.tif]
