# Supplementary material for: Humanin Protects RPE Cells from Endoplasmic Reticulum Stress-Induced Apoptosis by Upregulation of Mitochondrial Glutathione
Source: PLoS One. 2016 Oct 26;11(10):e0165150. doi: 10.1371/journal.pone.0165150 (PMC5081188; doi:10.1371/journal.pone.0165150)
Supplement: S1 Table — (PDF) [file pone.0165150.s006.pdf]

S1 Table

Gene	Forward	Reverse
Catalase	5'- GCC TGG GAC CCA ATT ATC TT-3'	5'-GAA TCT CCG CAC TTC TCC AG-3'
TRX-1	5'-TGT GGG CCT TGC AAA ATG A-3'	5'- GGA ATA TCA CGT TGG AAT ACT TTT CA- 3'
GRX-1	5'-TCG ATA TCA CAG CCA CCA AAC-3'	5'-CAC TGC ATC CGC CTA TAC AA-3'
GRX-2	5′-CTGGTTTGGAGCAGGAGCGGCTC-3'	5′-GCCTATGAGTGTCAGTTGCACC
SOD-II	5'-AATCAGGATCCACTGCAAGG-3'	5'- TAAGCGTGCTCCCACACAT-3'

Protein	Antibody	Source
Catalase	Rabbit anti-catalase	Abcam, Cambridge, MA
TRX-1	Rabbit polyclonal anti-TRX1	Santa Cruz Biotech, CA
GRX-1	Rabbit polyclonal anti-GRX1	GeneTex, Irvine, CA
GRX-2	Rabbit polyclonal anti-GRX2	GeneTex, Irvine, CA
SOD-II	Rabbit polyclonal anti-SOD2	Abcam, Cambridge, MA
